# Transcending Resolution Limits in HPLC and Diffusion NMR

**DOI:** 10.1021/acs.analchem.4c04418

**Published:** 2024-12-18

**Authors:** Nouran A. Hamed, Alexandria K. Shread, Gareth A. Morris, Mathias Nilsson

**Affiliations:** † Department of Chemistry, University of Manchester, Oxford Road, Manchester M13 9PL, United Kingdom; ‡ Department of Pharmaceutical Analytical Chemistry, Faculty of Pharmacy, Tanta University, Tanta 31111, Egypt; § Bruker UK Limited, Westwood Business Park, Longwood Cl, Coventry CV4 8HZ, United Kingdom

## Abstract

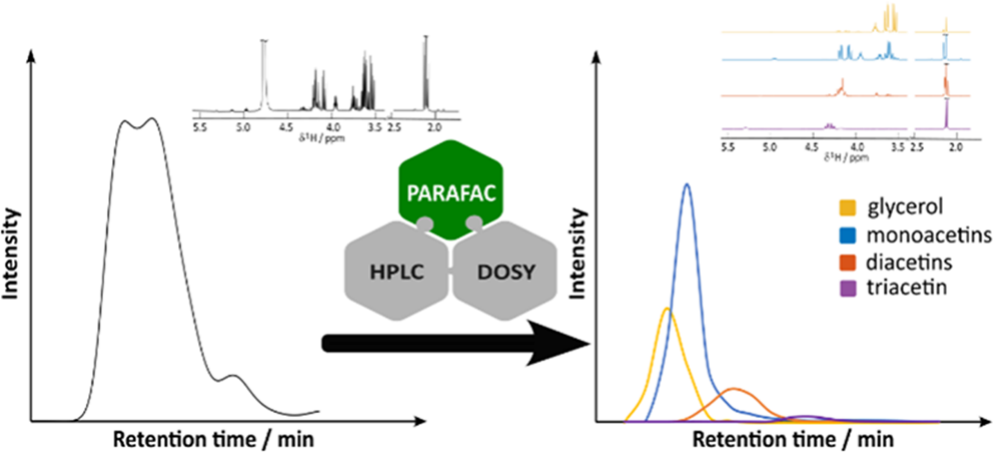

Mixture analysis is crucial in many areas of chemistry, and a wide variety of separation methods are in use. A common method for physical separation is high-performance liquid chromatography (HPLC), but resolution is a problem: chemically similar species coelute. An alternative approach is diffusion-ordered NMR spectroscopy (DOSY), in which the signals of mixture components are separated according to the diffusion coefficient. Again, separation is limited if species diffuse similarly or have overlap in their NMR spectra. Using the two techniques in combination can resolve both NMR spectra and the elution profiles of individual components, even where both techniques fail when used in isolation. Recording diffusion NMR data as a function of HPLC retention time gives a three-dimensional (3D) data set that can be analyzed using multiway statistical methods. PARAFAC analysis of diffusion NMR data measured from HPLC eluate for commercial “monoacetin” (a mixture of glycerol and its mono-, di-, and triacetates) yielded fully resolved and quantitative NMR spectra and elution profiles for all four components, whereas neither HPLC nor diffusion NMR applied independently was able to resolve the components.

## Introduction

A wide variety of analytical techniques and methods are available for the analysis of mixtures. One of the most commonly used is liquid chromatography, in particular high-performance liquid chromatography (HPLC), which can provide high sensitivity and precision in the separation and quantitation of mixture components. One important limitation, particularly in the separation of chemically similar species, is coelution: different components show similar elution times and hence overlapping peaks in a chromatogram. Obtaining well-resolved peaks is a potentially laborious task, which can involve varying any or all of the mobile phase composition, flow rate, stationary phase, temperature, and pH. HPLC method optimization can be time-consuming and generate large amounts of waste, which is not compatible with the practice of green analysis.^1^

An alternative technique for analyzing mixtures and unambiguously identifying components, which does not require physical separation, is nuclear magnetic resonance (NMR) spectroscopy. A central challenge in mixture analysis by NMR is distinguishing between the signals of the different species. Various NMR methods have been developed to facilitate compound identification within a mixture when the signals of different species overlap. Pure shift NMR simplifies spectra by collapsing the multiplicities of the signals.^2−5^ REST uses a combination of selective excitation and isotropic mixing to give the same relaxation weighting to each spin in a given spin system, allowing decomposition of the component subspectra.^6^ SCALPEL dissects NMR spectra using multiple parameters such as TOCSY evolution and relaxation, and then uses multivariate analysis to extract the spectra of individual spin systems.^7^ Targeted NMR methods using doubly selective excitation, or singlet states, can be effective for extracting component spectra in a single one-dimensional (1D) acquisition.^8,9^

Complementing the above information, diffusion NMR is one of the most effective and well-established methods for mixture analysis. Crucially, it allows the complete spectra of individual species to be distinguished and not just the spectra of individual coupled spin systems. The basic experiment encodes diffusion using a pulsed field gradient (PFG) spin or stimulated echo pulse sequence,^10^ in which gradient strength (*g*) is incremented while gradient pulse durations (δ) and the effective diffusion-encoding period (Δ′) are kept fixed. The attenuation of the signal intensities (*I*_g_) as a function of gradient amplitude is dependent on the rate of diffusion (and hence the size of the chemical species) during the diffusion-encoding period. The signal intensities can then be fitted to the Stejskal-Tanner eq (eq 1)^11^
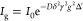
1where *I*_0_ is the signal intensity in the absence of diffusion, γ is the gyromagnetic ratio of the nuclear spins, and Δ′ is the diffusion-encoding time corrected for the effects of finite gradient pulse width, to obtain the diffusion coefficient values (*D*).

Different species can be distinguished by their different diffusion coefficients, with spectral data typically presented in a pseudo-2D DOSY (‘diffusion-ordered spectroscopy’) contour plot.^12−14^ Mixture components can be distinguished provided that their signals in the chemical shift dimension are resolved and that they have different diffusion coefficients. However, signal overlap, ubiquitous in more complex samples, materially limits the utility of such spectra. This is because extracting *D* using eq 1 assumes that a given signal arises from a single species, so overlapping signals from different species yield compromise values of the apparent diffusion coefficient. Unfortunately, it is not in general possible to distinguish the different contributions to a given signal decay unless the diffusion coefficients are very different.^15^

**Figure 1:**
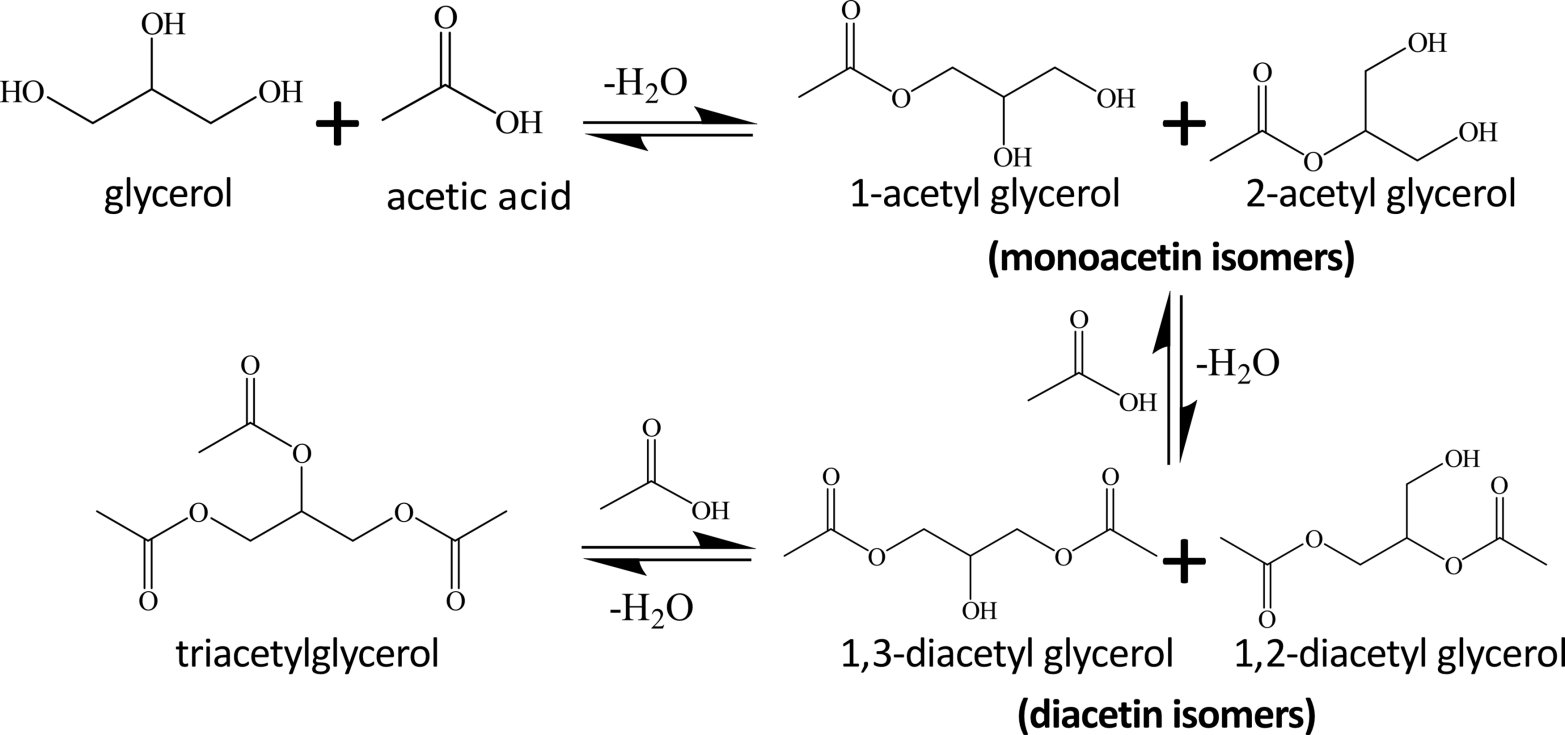
Mixture of acetins produced by esterification of glycerol with acetic acid.

NMR and HPLC can be combined to give HPLC-NMR, in which the NMR spectrometer serves as a very sophisticated online or off-line detector.^16^ This greatly increases the power of HPLC as a mixture analysis tool, but the fundamental issue of coeluting peaks remains (as it were) unresolved. Here we explore the combination of HPLC and diffusion NMR, showing that together, they make a much more powerful tool for mixture analysis. The HPLC-DOSY method is illustrated using commercial “monoacetin”, a mixture containing glycerol and its mono-, di-, and triacetates that has a wide range of applications, for example, in biodiesel. Technical grade monoacetin, produced by glycerol esterification with acetic acid (Figure 1), contains only about 50% of the two isomers of monoacetyl glycerol, the remainder being mostly glycerol and its di- and triesters (the two isomers of diacetin, and triacetin).^17,18^

Separation and analysis of the acetin mixture components have previously been carried out using gas chromatography^19−21^ and HPLC.^22,23^ Diffusion NMR has also been used with some success in the analysis of this mixture,^24^ but the DOSY spectrum only partially resolves the acetin signals (Figure S1), with overlap between proton signals leading to compromise apparent diffusion coefficients and preventing signal assignment to specific molecular species.

One way to disentangle overlapping signals in diffusion NMR is to acquire data for a range of different compositions, e.g., by acquiring diffusion NMR data during a chemical reaction, since this varies the contributions made by the individual spectra to the measured data.^25,26^ The resultant three-dimensional (3D) data set can be analyzed with powerful multiway statistical methods such as parallel factor analysis (PARAFAC),^27,28^ which exploits the independent variation in three (or more) dimensions to extract the NMR spectrum of each component.

Here, we propose an approach in which the overlap of the contributions of the acetin mixture components to the ultraviolet (UV) detected HPLC chromatogram and to the ^1^H NMR spectrum can be disentangled (together with the diffusion information for each component). Experimental measurements of diffusion-weighted NMR data as a function of retention time generate a data set *X* that varies in three orthogonal dimensions: chemical shift, retention time, and pulsed field gradient strength. Such a trilinear data set can be decomposed using the PARAFAC algorithm^26^ into contributions from *F* different mixture components (eq 2)
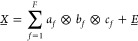
2where *a_f_*, *b_f_*, and *c_f_* are the signal dependences on the three independent variables for component *f*, and *E* is the residual.

In this work, we show that the combination of HPLC-DOSY and PARAFAC offers an effective solution to the problem of separating the contributions of individual mixture components to the experimental chromatogram and NMR spectra. In the absence of the equipment needed to record on-flow NMR spectra of the chromatographic eluate, the acquisition of experimental data was undertaken in two steps. First, a partial chromatographic separation was performed, in which a UV-detected chromatogram was recorded, and eluate fractions were collected for a range of retention times. These fractions were then evaporated and redissolved in D_2_O. Second, diffusion-weighted NMR spectra (loosely referred to as “DOSY data”) were then acquired for each fraction, using a series of different diffusion-encoding gradient strengths. The resultant 3D data set *X* was then decomposed with PARAFAC to give the NMR spectrum, diffusional decay, and elution profile for each of four components. The analysis requires only the data and the number of components *F* (here four), with no prior knowledge needed of the components’ chromatographic behavior, spectral properties, or hydrodynamic characteristics.

## Experimental Section

### Sample Preparation and Chromatographic Method

The chromatographic mobile phase used was a 10:90% v/v mixture of acetonitrile and deionized water, and the sample analyzed a 5% w/v solution of technical grade monoacetin (Acros Organics, Geel, Belgium) dissolved in the mobile phase. The partial chromatographic separation was performed on an Agilent HPLC 1260 infinity-II reversed-phase system equipped with a diode array detector and a semipreparative fraction collector. An Ascentis C18, 250 mm × 10 mm, column with a particle size of 5 μm was used. The separation was performed using ten identical runs with 3.5 mL/min flow rate, 206 nm detection wavelength, and a temperature of 35 °C. The injection volume was 50 μL, the run time was set to 7 min, and 21 fractions were pooled from the 10 runs. As some fractions contained only the mobile phase, only 16 of the fractions collected were employed in the remainder of the analysis.

### NMR Experiments

The collected HPLC fractions were dried in a vacuum desiccator to evaporate the solvents and redissolved in 0.6 mL of D_2_O containing 3.2 mM trimethylsilylpropanoate (TSP) as a reference. ^1^H DOSY measurements were carried out at 25 °C on a 400 MHz Bruker Avance III HD spectrometer equipped with a BBFO probe, using the Oneshot DOSY pulse sequence.^29^ The data were acquired with a spectral width of 6000 Hz (15 ppm); the transmitter offset frequency was set to 1360 Hz (3.4 ppm), and a time domain data length (TD) of 32,768 points was used. The experiment was performed with 16 gradient amplitudes in equal steps of gradient squared ranging from 4.8 to 38.5 G cm^–1^, using 16 transients, a recovery delay (D1) of 3 s, a total diffusion-encoding gradient pulse width of 2 ms, and a diffusion time of 0.1 s, in a total experiment time of ca. 28 min per sample.

### Data Processing

All data processing, including PARAFAC analysis, was performed using the General NMR Analysis Toolbox (GNAT),^30^ an open-source software package. None of the methods used are particularly demanding, and all can easily be performed on a standard PC or laptop. PARAFAC processing typically took less than 1 min. Zero-filling to 65,536 complex points was applied before Fourier transformation, followed by reference deconvolution^31,32^ using the TSP signal to a target line shape of a 1 Hz wide Lorentzian. The PARAFAC algorithm, using the N-way Toolbox^33^ implementation in GNAT, was employed to decompose the data into four components, using non-negativity constraints. The first gradient increment of the timecourse was excluded from the analysis because it is well-known for introducing errors related to instrumental imperfections. Since in this case the identities of the analytes were known, the normalization of the data modes extracted was adjusted so that the elution mode represented the relative molar concentration as a function of elution time of each component in the sample. This is not possible in a sample with unknown analytes, where an obvious choice would be to normalize the modes so that the elution mode shows the relative numbers of protons eluting (i.e., the relative signal integral) as a function of time. Normalization requires the measurement under quantitative conditions of a reference spectrum of a known species of known concentration, which then provides an absolute scale relating the signal integral to the proton concentration. The absolute concentrations of individual analytes can then be determined, where their identities are known, and the absolute proton concentration for each spectral peak can be determined where species have not been identified.

## Results and Discussion

### HPLC Analysis

The reversed-phase HPLC analysis of the acetin mixture with 90:10% v/v water:acetonitrile with UV detection at 206 nm showed similar retention of three of the mixture components, monoacetins, diacetins, and triacetin, which resulted in three overlapped chromatographic peaks. Glycerol could not be observed in the chromatogram, as it lacks the necessary chromophore. Although no resolved peaks are seen, the relative proportions of the different analytes evolve differently through the composite peak, with the analytes eluting, as expected, in descending order of polarity.

### NMR Analysis

**Figure 2:**
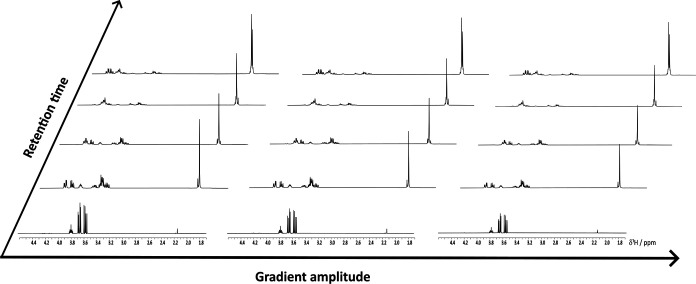
Subset of experimental spectra measured as a function of retention time and gradient amplitude, showing the change in eluate composition as a function of retention time and the NMR signal attenuation as a function of field gradient amplitude. The HPLC fractions selected here were the 8th, 12th, 14th, 16th, and 21st. For the diffusion decay, the spectra of the 1st, 3rd, and 5th gradient amplitudes are shown.

Figure 2 shows an excerpt from the 3D data set obtained by performing diffusion NMR experiments. The NMR signals vary in amplitude from fraction to fraction, reflecting the change in concentration of each species in the eluate as a function of retention time, and vary in amplitude as a function of the diffusion-encoding field gradient amplitude used, reflecting the small decreases in diffusion coefficient as the number of acetyl groups increases.

As was the case with the UV-detected chromatogram, DOSY alone was insufficient to separate the signals of the mixture components fully, as many of the signals overlap in the NMR spectrum.^24^ Multiway analysis of the 3D data set acquired was therefore performed.

### Multiway Analysis (PARAFAC)

**Figure 3:**
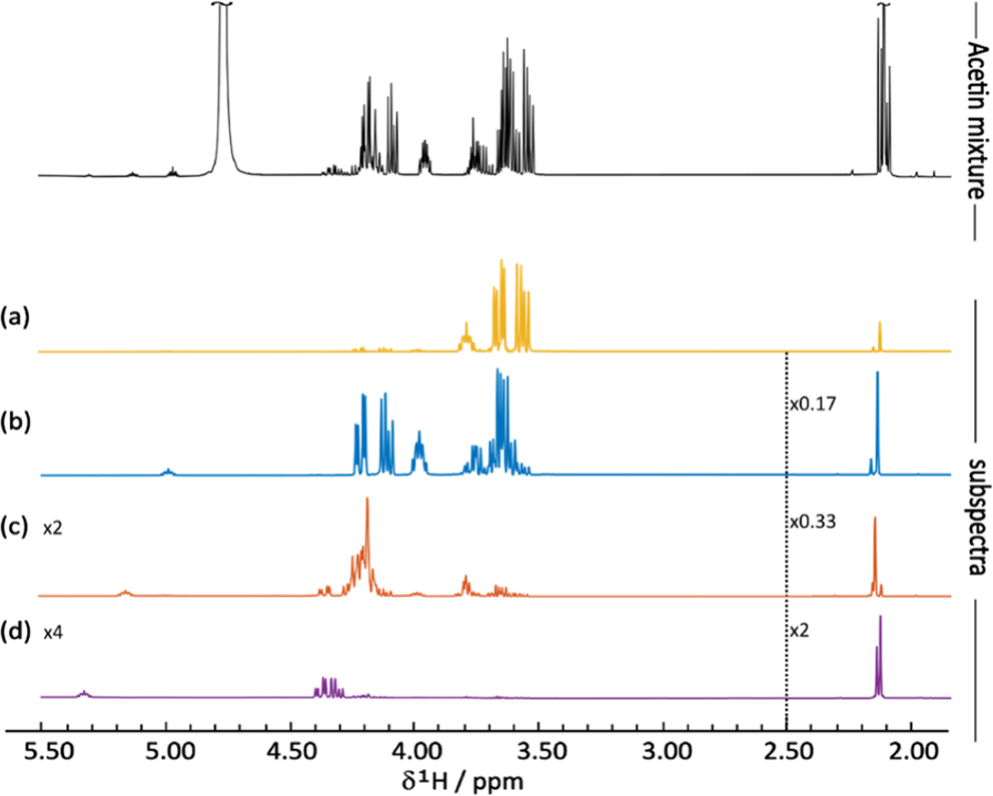
Experimental 1D ^1^H NMR spectrum of the acetin mixture (top) and the individual component subspectra obtained from PARAFAC for glycerol (a), monoacetins (b), diacetins (c), and triacetin (d). The water signal was removed from the subspectra by digital filtering. The raw data were acquired using a 400 MHz spectrometer.

**Figure 4:**
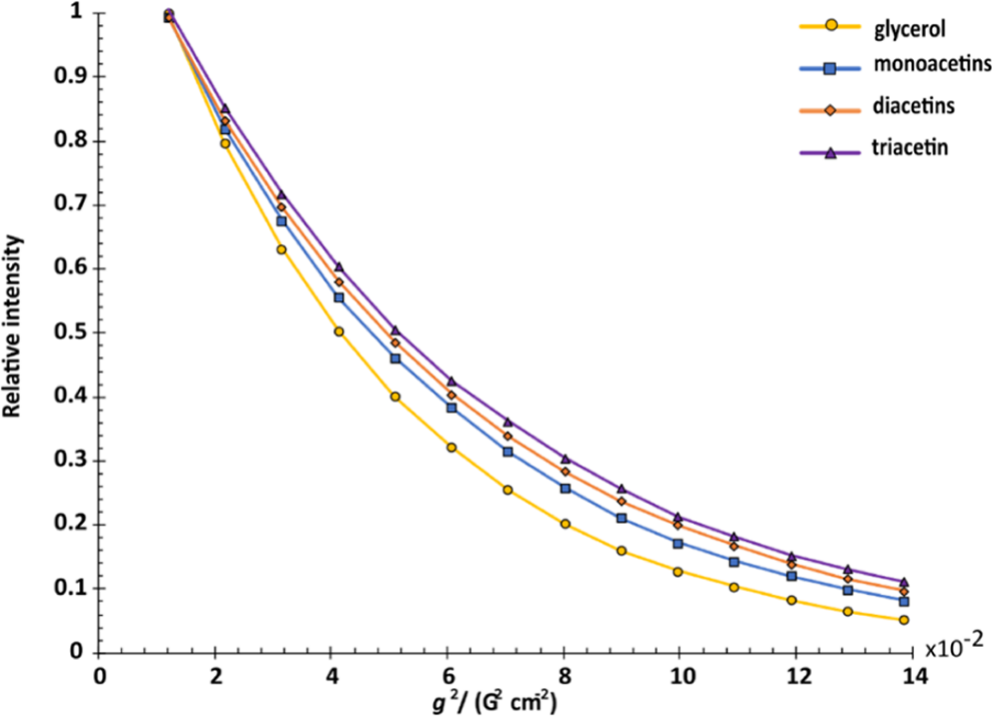
Diffusion attenuation profiles of the acetin mixture components obtained from PARAFAC.

PARAFAC analysis has three requirements: trilinearity (strictly multilinearity with a minimum of three dimensions), additivity, and variability.^34^ Trilinearity means that the chemical variation in each mode (dimension) of the data set is linear and represented by the same number of components; additivity means that the total signal is the linear superposition of the signals of those components; and variability means that each component varies differently from the others in each mode. In the proposed HPLC-DOSY method, this requires that the analytes have distinguishable spectra, distinguishable elution profiles, and distinguishable diffusion behavior. PARAFAC fitting was carried out to separate four components covering the whole NMR spectral region (2–5.5 ppm) apart from the water signal region around 4.7 ppm. Two of the signal modes yielded by PARAFAC are the 1D proton NMR spectra (Figure 3 spectra (a–d)) and the diffusional attenuation profiles (Figure 4) for the four components. Figure 3 shows excellent separation of the spectra, even in regions where there is severe signal overlap in the spectrum of the intact mixture. Table 1 lists the diffusion coefficients obtained by fitting the data of Figure 4 to the appropriate form of the Stejskal-Tanner equation (eq 1), showing the slight slowing of diffusion caused by each successive esterification.

**Table 1:** Diffusion Coefficients of the Acetin Mixture Components Obtained from PARAFAC Analysis

component	diffusion coefficient^a^/(10^–10^ m^2^ s^–1^)
glycerol	8.06 ± 0.04
monoacetin	7.00 ± 0.02
diacetin	6.55 ± 0.04
triacetin	6.01 ± 0.08

a Error limits quoted are twice the standard errors estimated in the fitting and do not include the effects of systematic errors such as temperature calibration and/or variation.

**Figure 5:**
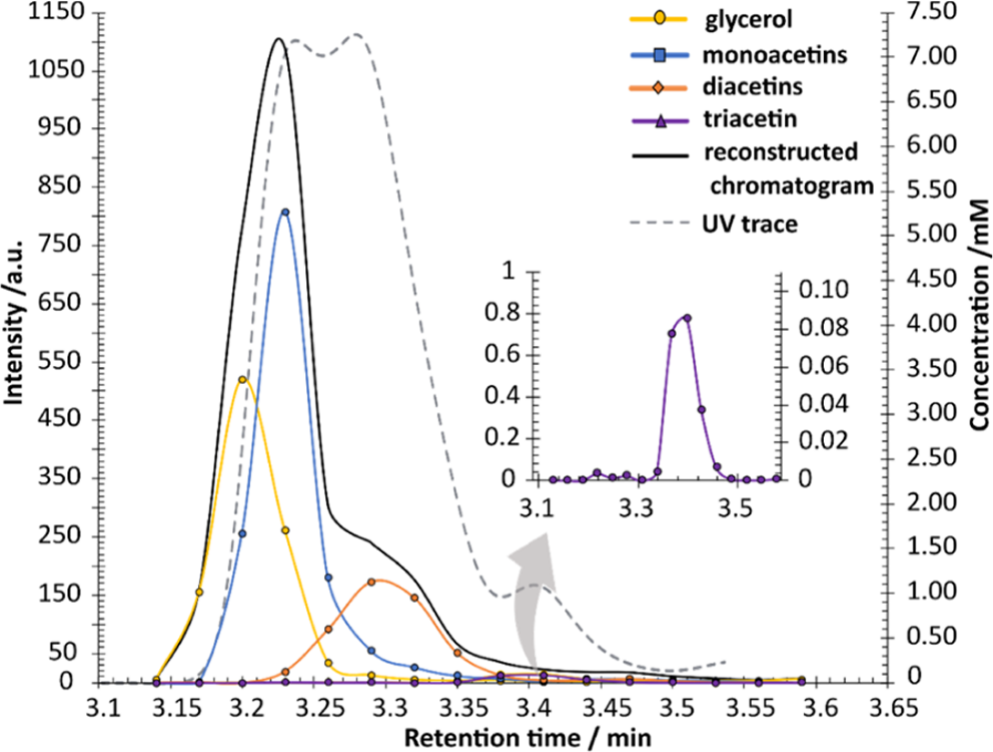
Comparison between the directly UV-detected (broken line, left scale) and NMR (solid lines, right scale) chromatograms for HPLC experiments on commercial “monoacetin”. The NMR chromatograms were obtained by PARAFAC decomposition of diffusion-weighted NMR data from pooled fractions; the solid lines are smoothed between the original data points and shown as dots. The sum of the individual PARAFAC chromatograms, which reconstruct a complete NMR-detected chromatogram, is shown in black. Unlike the UV chromatogram, this summed trace directly reflects the molar concentrations of the four species and includes the signal of glycerol, which is absent from the UV trace as it lacks the necessary chromophore. The right-hand scale indicates the component concentrations in the pooled aliquots measured, so the data points shown sum to approximately 5/6 of the concentrations of the four species in the original analyte solution (10 × 50 μL injections, fractions pooled and made up to 600 μL).

The third mode in the PARAFAC output is the relative concentration timecourse for each species over the elution time range spanned by the fractions collected. Figure 5 compares these timecourses with the UV detector output recorded during the chromatographic separation (the broken gray line). Note that glycerol does not contribute to the UV absorbance since it lacks an appropriate chromophore. With the choice of normalization used, the NMR timecourses are directly proportional to the molar concentrations of the four species rather than being scaled by molar absorption coefficient at 206 nm. The solid black line in Figure 5 shows the total eluate molar concentration for the four components; as expected, the actual concentrations of di- and triacetin are lower compared to monoacetin than is suggested by the UV trace because they have, respectively, two and three carbonyl chromophores rather than one.

As can be seen from Figures 3, 4, and 5, PARAFAC was successful in resolving four components of the acetin mixture: glycerol, monoacetins, diacetins, and triacetin. While a significant victory, this is of course only half the battle where this sample is concerned: while the combination of HPLC-DOSY and PARAFAC can separate mono- and diacetin, it cannot distinguish between their different isomers. These will have almost identical diffusion coefficients; while they may differ slightly in retention time, there is no experimental evidence for this. This method is applicable only to compounds that exhibit detectable differences in both retention and diffusion. If the compounds under investigation show the same diffusion, then another property such as relaxation would be preferred, as used in the REST^6^ and SCALPEL^7^ experiments.

The work described in this paper uses separate HPLC and NMR instruments to demonstrate a proof of concept. Clearly, for practical applications, dedicated HPLC-NMR equipment, which was not available for the work described, would be greatly preferable, allowing for much faster and more reproducible implementation. Instead of the fraction collection and drying steps, data would be acquired directly in on-flow, stop-flow, or loop-storage mode. The latter two are appropriate for dilute samples as they allow time averaging to improve the signal-to-noise ratio. For on-flow measurements, a flow-compensated DOSY pulse sequence should be used to avoid the effects of velocity-dependent phase shifts^35^

## Conclusions

Adding a third dimension to diffusion data, such as a coherent change in the relative concentrations of partially resolved mixture constituents during a chromatography run, creates a 3D data set that can be analyzed with a multiway method such as PARAFAC to extract component spectra. With no prior knowledge of the actual behavior in the three studied modes, PARAFAC decomposition worked successfully here even with significant spectral overlap, small differences in diffusion coefficient, and a range of concentrations of mixture components. Only in extremis would this proof of principle, where fractions were collected, dried down, dissolved, and analyzed by NMR, be a practical tool for mixture analysis. However, it gives good grounds for optimism that experiments using online NMR detection, e.g., using stopped flow or loop storage, will provide similar gains. Provided that appropriate pulse sequence design is used to refocus the effects of bulk flow in diffusion-encoding experiments, it should also be possible to carry out experiments on flow, e.g., adapting ultrafast NMR methods.^35,36^ The present method relies on differences in the diffusion coefficient, and although the PARAFAC approach works remarkably well with very small differences in *D*(7), there are other NMR parameters such as relaxation and evolution times that can also be used to good effect.^6,7^ We postulate that the novel approach presented in this work has the potential to become a valuable tool for complex mixture analysis.

## Supporting Information Available

Assignments of the ^1^H NMR subspectra of the commercial “monoacetin” sample, a DOSY spectrum of the mixture, and the pulse sequence code used (PDF)

## Abbreviations

NMR: nuclear magnetic resonance
HPLC: high-performance liquid chromatography
REST: relaxation-encoded selective TOCSY
SCALPEL: spectral component acquisition by localized PARAFAC extraction of linear components
DOSY: diffusion-ordered spectroscopy
PARAFAC: parallel factor analysis
UV: ultraviolet
TSP: trimethylsilyl propionate
