# Supplementary material for: Transcending Resolution Limits in HPLC and Diffusion NMR
Source: Anal Chem. 2024 Dec 18;96(52):20475–80. doi: 10.1021/acs.analchem.4c04418 (PMC11696829; doi:10.1021/acs.analchem.4c04418)
Supplement: Supplementary file 1 — ac4c04418_si_001.pdf [file ac4c04418_si_001.pdf]

Transcending resolution limits in HPLC and diffusion NMR

Supporting Information

Nouran A. Hamed, Alexandria K. Shread, Gareth A. Morris, and Mathias Nilsson

Contents

DOSY spectrum of the acetin mixture 1

Assignments of the acetin mixture 2

Pulse sequence code (dosyoneshot) 3

AU-program (UoMdosy) 6

DOSY spectrum of the acetin mixture

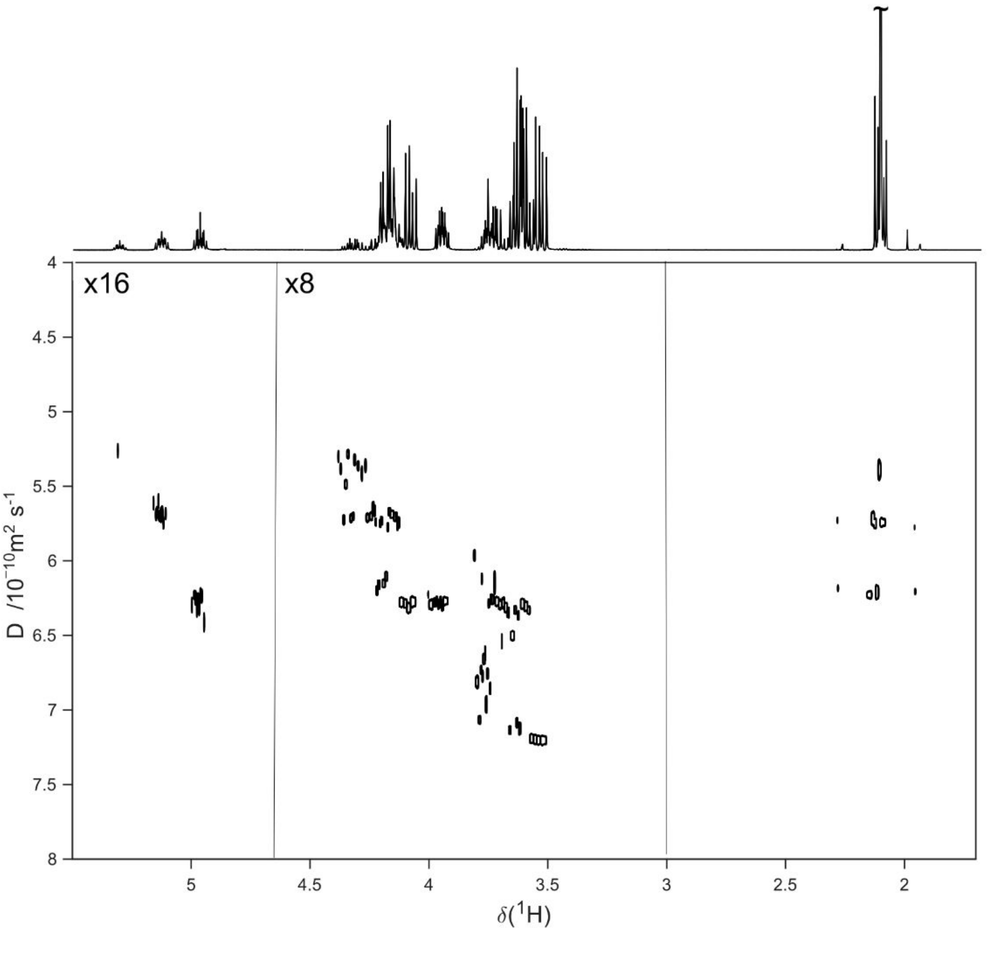

Figure S1. 400 MHz ^1H DOSY spectrum of 1.34 g% commercial 'monoacetin' in D_2O; the residual water signal was removed by digital filtering. This spectrum was obtained using a 400 MHz Bruker Avance III HD spectrometer at 25°C using 32 gradient increments ranging from 4.8 to 38.5 G cm^{-1}.

[1]H assignments of the acetin mixture

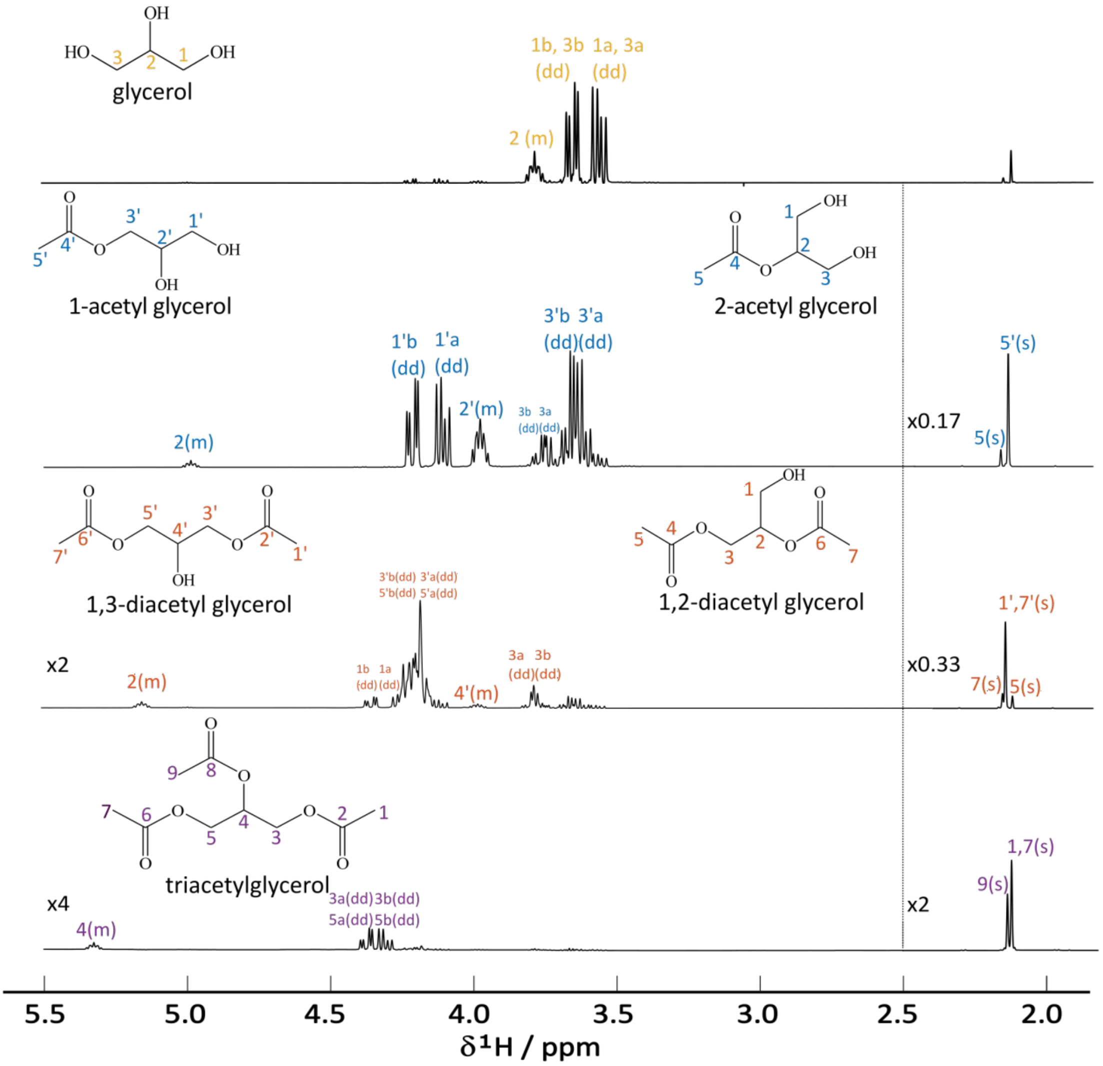

Figure S2. ^{1}H subspectra obtained by PARAFAC decomposition of experimental data for commercial ‘monoacetin’ in D_2O acquired as described in the main text, showing the assignments of the mixture components. The water signal was removed by digital filtering.

Pulse sequence code (dosyoneshot)

```
; Ralph Adams, Juan Aguilar, Robert Evans, Mathias Nilsson and Gareth Morris
; University of Manchester
; Release 1.0c (27Mar2012)

; Source citation:
; M.D. Pelta, G.A. Morris, M.J. Stchedroff, S.J. Hammond, Magn. Reson. Chem. 40 (2002) S147-S152.

; Other relevant papers that could be of use include:
; A. Botana, J.A. Aguilar, M. Nilsson, G.A. Morris, J. Magn. Reson. 208 (2011) 270-278.

;2D Doneshot pulse sequence
;$CLASS=HighRes
;$DIM=2D

#include <Avance.incl>
#include <Grad.incl>
#include <Delay.incl>

define list<gradient> diff=<Difframp>

"cnst14=0.2"
"cnst15=1+cnst14"  ; 1 + alpha
"cnst16=1-cnst14"  ; 1 - alpha

"p2=p1*2"

"cnst17=(2*p1+d16)*0.000001"; Dtau

;Assuming square gradient pulses
;"cnst18=0.000001*p30*2*0.000001*p30*2*(d20+(0.000001*p30/3)*(cnst14*cnst14-
2)+((cnst17)/2)*(cnst14*cnst14-1))" ; Dosytimecubed

;Assuming half-sine gradient pulses [most common on Bruker systems]
"cnst18=0.000001*p30*2*0.000001*p30*2*(d20 - (2*0.000001*p30*(5-3*cnst14*cnst14)/16) - (cnst17*(1-
cnst14*cnst14)/2) )" ; Dosytimecubed

"DELTA1=d20-4.0*p1-4.0*p30-5.0*d16-p19"

"acqt0 = 0"
baseopt_echo

1 ze
2 30m pl1:f1
50u BLKGRAD
  50u LOCKH_OFF
  d1
  50u LOCKH_ON
  50u UNBLKGRAD

   p19:gp2*-1.0                    ;Spoiler gradient balancing pulse
  d16
  p1 ph1                           ;1st 90
  p30:gp1*diff*cnst16                         ;1 - alpha
  d16
   p2 ph2                          ;First 180
  p30:gp1*-1*diff*cnst15                      ;1 + alpha
  d16
  p1 ph3                           ; 2nd 90
  p30:gp1*diff*2*cnst14                       ;Lock refocusing pulse pulse
  d16
  p19:gp2                          ;Spoiler gradient balancing pulse
```

```
 d16
 DELTA1
 p30:gp1*diff*2*cnst14                  ;Lock refocusing pulse pulse
 d16
 p1 ph4                                 ; 3rd 90
 p30:gp1*diff*cnst16                            ; 1 - alpha
 d16
 p2 ph5
 p30:gp1*-1*diff*cnst15                         ;1 + alpha
 d16 BLKGRAD
 go=2 ph31
 30m mc #0 to 2 F1QF(igrad diff)
 50u LOCKH_OFF
exit

;phase cycling
ph1 = 0 0 0 0 0 0 0 0 2 2 2 2 2 2 2 2
ph2 = 0
ph3 = 0 0 0 0 2 2 2 2
ph4 = 0 1 2 3
ph5 = 0 0 0 0 0 0 0 0 0 0 0 0 0 0 0 0 2 2 2 2 2 2 2 2 2 2 2 2 2 2 2 2
ph31= 0 3 2 1 2 1 0 3 2 1 0 3 0 3 2 1

;pl1           f1 channel - power level for pulse (default)
;p1            f1 channel -  90 degree high power pulse
;p19           gradient pulse 2 (spoil gradient)
;p30           gradient pulse (little DELTA*0.5)
;d1            relaxation delay; 1-5 * T1
;d16           delay for gradient recovery
;d20           diffusion time (big DELTA)
;gp1           diffusion encoding gradient
;gp2           Oneshot CTP gradients
;cnst14        alpha, typically 0.2
;cnst15        1+alpha
;cnst16        1-alpha
;cnst17        Dtau
;cnst18        dosytimecubed
;NS            1 * n
;DS            1 * m
;td1           number of experiments
;FnMODE                QF
; use  gradient value  gpz1: 100  and  gpz7 : 100
;use AU-program UoMdosy to calculate gradient ramp-file Difframp
;use xf2 and DOSY processing

;$Id: Doneshot,v 1.0b 2011/11/30 Copyright University of Manchester
```

AU-program (UoMdosy)

This AU-program is a slightly modified version of the standard dosy macro in the Topspin software package.

```
/*** ^^A -*-C++-*- **********************************************/
/*  dosy          29.01.2018                          */
/****************************************************************/
/*  Short Description :                               */
/*  AU program to setup diffusion/DOSY experiments             */
/****************************************************************/
/*  Keywords :                                      */
/*  diffusion, DOSY                                   */
/****************************************************************/
/*  Description/Usage :                                */
/*  This AU program creates a JCAMP gradient ramp file          */
/*  called "Difframp", which is stored in "..../lists/gp".     */
/*  The arguments are the start value for the ramp, the         */
/*  final value, the number of steps, whether a linear (l),     */
/*  "squared" (q) or exponential (e) ramp. All parameters may be passed   */
/*  on the command line. example:    xau UoMdosy 2 98 16 l   */
/*  This will create a ramp starting with 2% amplitude,         */
/*  ending with 98% in 16 linearly spaced steps */
/*  In addition a file called "difflist" is created in the      */
/*  expno directory, which contains the gradient amplitudes     */
/*  scaled to rectangular gradients in G/cm as a kind of       */
/*  status parameter. This file is needed for the              */
/*  processing. It uses the information in the file            */
/*  XWINNMRHOME/conf/instr/gradient_calib to calculate          */
/*  the correct gradient strength. The gradient strength        */
/*  scaling factor can be set using 'setpre'.              */
/*  Some parameters are checked for consistency.               */
/*  In the case of the so called "squared" ramp, the           */
/*  gradient amplitudes follow a square root function          */
/*  leading to a linear response of the signal amplitudes.      */
/*  A new gradient shape "Difftrap" (a trapezoidal shape) is    */
/*  now supported. This is a nearly rectangular shape with      */
/*  rise and fall times of 50 us. This is automatically         */
/*  calculated if GPNAM6 is Difftrap.                  */
/****************************************************************/
AUERR = dosy(curdat);
QUIT

#include <ShapeIO/ShapeIOC.h>
#include <pstruc.h>
#include <pstruc_preemp.h>

/*  Define some constants and variables         */
#define MAX_POINTS  4096
#define TLL 1024
const   int debugflag=0;
const   float gammaH=4257.74691;

static  int getshapescale(const char*, double*);
static  int scale_gradshape(const char*, double);
static  int trev_gradshape(const char*);
static  struct preemp_pars preempPars =
#include <pinit_preemp.h>


int dosy(const char* curdat)
{
int     diffpoints=0, restore=0, fnmod1, fnmod2=0, fnmod3, pmod, nShapePoints, ret;
```

```
float   gstart, gstop;
double  shapescale=0.63662, gcalib=53.5, minAmp, maxAmp, integFac;
double* phPtr;
double* value;
char    text[TLL], path[PATH_MAX], probeId[100], errorBuf[256];
char    rampname[TLL], shapename[TLL], stcmd[TLL];
const   char* ramptype = "l";
const   char* answer = "n";
const   char* rga = "n";
const   char* nospool = "n";
FILE*   fptr;
#ifndef   HAS_WINDOWS_FEATURES
mode_t  cmask;
#endif

FETCHPAR("PARMODE",&pmod)
if ( (pmod < 1) || (pmod > 3) )
   STOPMSG("Not a 2D, 3D or 4D dataset");

if (pmod == 1)      /* 2D */
   {
   FETCHPAR1("FnMODE",&fnmod1)
   if (fnmod1 != 1)
      STOPMSG("FnMODE must be QF in diffusion dimension (F1)")
   FETCHPAR1("TD",&diffpoints)
   }

if (pmod == 2)      /* 3D */
   {
   FETCHPAR1("FnMODE",&fnmod2);        /* F2 */
   FETCHPAR3("FnMODE",&fnmod1);        /* F1 */
   if ( (fnmod1 != 1) && (fnmod2 != 1) )
      STOPMSG("FnMODE must be QF in diffusion dimension")

   if (fnmod2 == 1)    { FETCHPAR1("TD",&diffpoints) }
   else                { FETCHPAR3("TD",&diffpoints) }
   }


if (pmod == 3)      /* 4D */
   {
   FETCHPARN(3,"FnMODE",&fnmod3);        /* F3 */
   FETCHPARN(2,"FnMODE",&fnmod2);        /* F2 */
   FETCHPARN(1,"FnMODE",&fnmod1);        /* F1 */
   FETCHPARN(3, "TD", &diffpoints)
   }


/*  get shapescale value    */
FETCHPAR("GPNAM 1",shapename)

if (strcmp(shapename,"Difftrap") == 0)
   {
   XCMD("difftrap")
   ERRORABORT
   }

if (getshapescale(shapename, &shapescale) < 0)  return -1;


/*  evaluate command line or ask for parameters     */
if (i_argc > 2  &&  strcmp(i_argv[2], "restore") == 0)
   restore = 1;
```

```
if (i_argc <= 5  &&  restore == 0)
   {
   gstart=10;
   gstop=80;
   Proc_err(DEF_ERR_OPT,"Define the gradient ramp for shape\n%s (Integral = %.3f)",
       shapename, shapescale);
   GETFLOAT("Enter first gradient amplitude:  ", gstart )
   GETFLOAT("Enter final gradient amplitude:  ", gstop )
   GETINT("Enter number of points:", diffpoints )
   strcpy(rampname, ramptype);
   GETSTRING("ramp type (l q e {linear/squared/exponential} ):  ", rampname )
   ramptype = rampname;
   answer = "q";
   }
else
   {
   if (restore)
      {
      FETCHPAR("CNST 20",&gstart)
      FETCHPAR("CNST 21",&gstop)
      if (gstop < 0)
         {
         gstop = -gstop;
         if (gstart < 0)
            {
            gstart = -gstart;
            ramptype = "e";
            }
         else
            {
            ramptype = "q";
            }
         }
      else
         {
         ramptype = "l";
         }

      Proc_err(DEF_ERR_OPT,
         "Gradient ramp (%s) from %.1f to %.1f in %d steps restored",
         ramptype,gstart,gstop,diffpoints);
      }
   else
      {
      gstart = atof(i_argv[2]);
      gstop  = atof(i_argv[3]);
      diffpoints = atoi(i_argv[4]);
      ramptype = i_argv[5];

      if (i_argc > 6)
         answer = i_argv[6];
      if (i_argc > 7)
         rga = i_argv[7];
      if (i_argc > 8)
         nospool = i_argv[8];
      }
   }


/*  Check for legal number of points    */
if ((gstart > gstop) ||
   (gstop > 100) ||
```

```
   (diffpoints <= 0) ||
   (diffpoints > MAX_POINTS))
   STOPMSG("illegal arguments")


/*  calculate ramp and store shape using shapetool  */
switch(ramptype[0])
   {
   case 'l' :  {
            sprintf(stcmd,"st generate Ramp %d false %.1f %.1f filename=Difframp", diffpoints, gstart, gstop);
            XCMD(stcmd)
            ERRORABORT
            sprintf(stcmd,"st generate Ramp %d false %.1f %.1f filename=DifframpR", diffpoints, gstop, gstart);
            XCMD(stcmd)
            ERRORABORT

            STOREPAR("CNST 20", gstart)
            STOREPAR("CNST 21", gstop)
            sprintf(text,"lin G (%d points)",diffpoints);
            STOREPAR("EXP",text)
            }
            break;

   case 'q' :  {
            sprintf(stcmd,"st generate QRamp %d false %.1f %.1f filename=Difframp", diffpoints, gstart, gstop);
            XCMD(stcmd)
            ERRORABORT
            sprintf(stcmd,"st generate QRamp %d false %.1f %.1f filename=DifframpR", diffpoints, gstop, gstart);
            XCMD(stcmd)
            ERRORABORT

            STOREPAR("CNST 20", gstart)
            STOREPAR("CNST 21", -gstop)
            sprintf(text,"lin Q (square, %d points)",diffpoints);
            STOREPAR("EXP",text)
            }
            break;

   case 'e' :  {
            sprintf(stcmd,"st generate Efunc %d false %f filename=Difframp", diffpoints, gstart*100.0/gstop);
            XCMD(stcmd)
            ERRORABORT
            if (scale_gradshape("Difframp", gstop) < 0)   return -1;
            if (trev_gradshape("Difframp") < 0)   return -1;

            sprintf(stcmd,"st generate Efunc %d false %f filename=DifframpR", diffpoints, gstart*100.0/gstop);
            XCMD(stcmd)
            ERRORABORT
            if (scale_gradshape("DifframpR", gstop) < 0)   return -1;

            STOREPAR("CNST 20", -gstart)
            STOREPAR("CNST 21", -gstop)
            sprintf(text,"exp G (%d points)",diffpoints);
            STOREPAR("EXP",text)
            }
            break;

   default  :  STOPMSG("illegal ramp type")
   }


/*  read gradient ramp values (just created by stcmd) for conversion to gradient strength    */
if (getParfileDirForRead("Difframp", GP_DIRS, path) < 0)
```

```
{
   Proc_err(DEF_ERR_OPT, "%s: %s", "Difframp", path);
   return -1;
}

value = readShapeC(path, &nShapePoints, &phPtr, &minAmp, &maxAmp, &integFac);
if (value == 0)
   Proc_err(DEF_ERR_OPT,"cannot open ramp file for reading\n%s", path);


#if 0
/*  get gradient calibration    */
sprintf(path, "%s/conf/instr/gradient_calib", PathXWinNMRInst());
if ((fptr = fopen(path, "r")) != 0 )
   {
   char unit[TLL];
   if (fscanf(fptr, "%lf %s", &gcalib, unit) == 2)
      {
      if (strcmp(unit,"G/cm") != 0)
         {
         if (strcmp(unit,"G/mm") == 0)
            gcalib*=10;
         else
            gcalib=49.9;
         }
      if (answer[0] != 'y' && gcalib <= 10.0)
         {
         Proc_err(ERRORH_OPT, "gradient calibration may be wrong\n%.4f G/cm", gcalib);
         }
      }
   else
      {
      gcalib=49.9;
      }
   fclose( fptr );
   }
#endif

/* get local copy of preemp default file */
getProbeId(probeId, sizeof(probeId));
sprintf(path, "%s/instr/curprobepreempdefault", PathXWinNMRConf());
#ifndef   HAS_WINDOWS_FEATURES
cmask = umask(0);
#endif
ret = getPreempDefaultFileForProbe(probeId, path, errorBuf, sizeof(errorBuf));
#ifndef   HAS_WINDOWS_FEATURES
umask(cmask);
#endif

if (ret < 0)
{
   /* the copy process failed */
   gcalib = 49.9;
}
else
{
   /* retrieve gradstrength from the local copy of the default-file */
   ret = getpar(path, "GRADCC", &preempPars);

   if (ret < 0)
   {
      /* something went wrong in getpar */
      gcalib = 53.5;
```

```
    }
    else
    {
       gcalib = preempPars.GRADCC / gammaH;
    }
}


/*  open difflist file    */
if ((fptr = fopen(ACQUPATH("difflist"), "wt")) == 0)
   STOPMSG("Cannot create difflist")


/*  store difflist with gradient strength values        */
TIMES(diffpoints)
   fprintf(fptr, "%.3f\n", value[loopcount1] * shapescale * gcalib);
END

deleteShapeC();
fclose(fptr);

STOREPAR("GPZ 1",100.0)


if (pmod == 1)  { STOREPAR1("TD",diffpoints) }
if (pmod == 2)
   {
   if (fnmod2 == 1) { STOREPAR1("TD",diffpoints) }
   else             { STOREPAR3("TD",diffpoints) }
   }
if (pmod == 3)  { STOREPARN(3,"TD",diffpoints); }

Show_meta(SM_RAWP);

if (i_argc <= 2)
   {
   Proc_err(DEF_ERR_OPT, "Gradient ramp from %.1f to %.1f written", gstart, gstop);
   }

return 0;
}


/* subroutines *************************************************************/
static int getshapescale(const char* shapename, double* scal)
{
char    path[PATH_MAX];

if (getParfileDirForRead(shapename, GP_DIRS, path) < 0)
   {
   Proc_err(DEF_ERR_OPT, "%s: %s", shapename, path);
   return -1;
   }

*scal = getIntegFacC(path);

if (debugflag > 0) {
   Proc_err(ERROPT_AK_OK,"DEBUG\nshapename = %s\nshape integfactor = %.2f", shapename, *scal); }

return 0;
} /* end subroutine */
```

```
static int scale_gradshape(const char* gradname, double fact)
{
const   double *inputData;
const   double *phaseData = 0;
int     size, mode;
char    fname[PATH_MAX],text[TLL];

if (getParfileDirForRead(gradname, GP_DIRS, fname) < 0)
   {
   Proc_err(DEF_ERR_OPT, "%s: %s", gradname, fname);
   return -1;
   }

sprintf(text, "%f, false", fact);

inputData = manipulateShapeC(fname, "scale", text, 0, &phaseData, &size, &mode);
if (inputData)
   {
   if (writeShapeC(fname, inputData, size, 0, 0, 0) == 0)
      {
      Proc_err(DEF_ERR_OPT, "write gradient ramp file failed:\n%s", fname);
      ABORT
      }
   }

return 0;
} /* end subroutine */


static int trev_gradshape(const char* gradname)
{
const   double *inputData;
const   double *phaseData = 0;
int     size, mode;
char    fname[PATH_MAX],text[TLL];

if (getParfileDirForRead(gradname, GP_DIRS, fname) < 0)
   {
   Proc_err(DEF_ERR_OPT, "%s: %s", gradname, fname);
   return -1;
   }

sprintf(text, "1.0, false");

inputData = manipulateShapeC(fname, "trev", text, 0, &phaseData, &size, &mode);
if (inputData)
   {
   if (writeShapeC(fname, inputData, size, 0, 0, 0) == 0)
      {
      Proc_err(DEF_ERR_OPT, "write gradient ramp file failed:\n%s", fname);
      ABORT
      }
   }

return 0;
} /* end subroutine */
```
